# AGING WITH HIV: ASSOCIATION OF ART REGIMEN AND ADHERENCE TO VIRAL SUPPRESSION

**DOI:** 10.1093/geroni/igad104.2917

**Published:** 2023-12-21

**Authors:** Jasmine Manalel, Jennifer Kaufman, Ethan Fusaris, Arlene Correa, Jerome Ernst, Mark Brennan-Ing

**Affiliations:** Hunter College, New York City, New York, United States; Brookdale Center for Healthy Aging, Hunter College, City University of New York, New York City, New York, United States; Amida Care, New York, New York, United States; Brown University, Providence, Rhode Island, United States; Amida Care, New York, New York, United States; Hunter College, CUNY, New York City, New York, United States

## Abstract

Adherence to antiretroviral therapy (ART) is essential to effective management of HIV among an increasing population of older adults. Newer ART regimens are more “forgiving” of poor adherence allowing for viral suppression at lower adherence levels (i.e., < 90%). This is especially promising for some older adults who may struggle with adherence due to high rates of multimorbidity and polypharmacy. We aimed to determine how ART forgiveness varies depending on ART type and adherence. Data came from 2017-2019 claims and clinical records of 1,044 HIV-positive members of a Medicaid managed care plan who were 50 to 65 years of age. Pharmacy fill data were examined on a quarterly basis to characterize ART medications using latent class analysis (LCA), which captures the complexity of real-world ART usage (i.e., multiple medications, ART switching). LCA yielded five distinct ART utilization patterns over three years, mostly characterized by recent medications and formulations of ART, though they varied in number of tablets and in medication class (e.g., boosted protease inhibitors). Logistic regression models adjusting for demographics and health-related covariates revealed that ART medication patterns did not consistently differ in odds of maintaining viral suppression across adherence levels (< 50%, 50% to < 80%, 80% to < 85%, 85% to < 90%, and >=90%). In contrast, other factors, including nadir CD4 count and behavioral health conditions, were related to viral suppression across models. This highlights the importance of life-course illness trajectories when considering disease management and treatment strategies for older adults living with HIV, especially those with difficulty maintaining near-perfect adherence.

